# IMPROVING KNOWLEDGE OF LGBTQ+ AGING THROUGH A QUEERING GERONTOLOGY CAMPAIGN

**DOI:** 10.1093/geroni/igad104.2028

**Published:** 2023-12-21

**Authors:** Austin Oswald, Lujira Cooper, Leo O’Driscoll, Nancy Giunta

**Affiliations:** University of Washington, Seattle, Washington, United States; Services and Advocacy for GLBT Elders, New York City, New York, United States; Silberman School of Social Work, Hunter College, CUNY, New York City, New York, United States; Silberman School of Social Work, Hunter College, CUNY, New York City, New York, United States

## Abstract

The marginalization of lesbian, gay, bisexual, transgender, and queer plus (LGBTQ+) content in health and human services education is well documented and varies considerably due to instructors’ attitudes as well as government and institutional policies intended to censor conversations about sexual orientation and gender identity. Lack of LGBTQ+ content in the curriculum may require creative solutions to fill the gaps in mainstream education which often goes unchallenged due to dominant heteronormative theory, literature, and teaching. Without adequate training, service providers may not have sufficient knowledge and skills for competent practice with LGBTQ+ older adults. This presentation bridges critical pedagogy with emancipatory gerontology to reflect upon a student led “queering gerontology” campaign to raise awareness about LGBTQ+ aging. Applying principles from participatory action research, the presenters analyze personal narratives in combination with program evaluation data to describe our process conceptualizing, implementing, and evaluating the campaign. We found that students are eager to learn about LGBTQ+ aging experiences and have innovative ideas to close the gaps in knowledge caused by heteronormative educational contexts. Supporting student led learning, intersectional coalition building, and intergenerational wisdom sharing were central to combatting programmatic and institutional constraints that limit LGBTQ+ content in the curriculum. The presentation concludes by highlighting future teaching and research directions that support students in their pursuit of knowledge while challenging heteronormative structures of higher education.

